# Clinical and microbiological characteristics, and impact of therapeutic strategies on the outcomes of children with candidemia

**DOI:** 10.1038/s41598-017-01123-6

**Published:** 2017-04-24

**Authors:** Ming-Horng Tsai, Jen-Fu Hsu, Shih-Ming Chu, Pey-Jium Chang, Mei-Yin Lai, I-Hsyuan Wu, Hsuan-Rong Huang, Ming-Chou Chiang, Ren-Huei Fu, Jang-Jih Lu

**Affiliations:** 1 Division of Neonatology and Pediatric Hematology/Oncology, Department of Pediatrics, Chang Gung Memorial Hospital, Yunlin, Taiwan; 2 Division of Pediatric Neonatology, Department of Pediatrics, Chang Gung Memorial Hospital, Taoyuan, Taiwan; 3 Department of Laboratory Medicine, Chang Gung Memorial Hospital at Linkou, Taoyuan, Taiwan; 4 College of Medicine, Chang Gung University, Taoyuan, Taiwan; 5 Department of Medical Biotechnology and Laboratory Science, Chang Gung University, Taoyuan, Taiwan; 6 Department of Medicine, Graduate Institute of Clinical Medical Science, College of Medicine, Chang Gung University, Taoyuan, Taiwan

## Abstract

We aimed to determine the clinical and microbiological characteristics of *Candida* bloodstream infections in children and the impact of therapeutic strategies on outcomes. All pediatric patients with candidemia from a medical center in Taiwan over a 13-year period (2003–2015) were included and a total of 262 patients with 319 episodes of candidemia were analyzed. Overall susceptibility to fluconazole was 86.1%. Cumulative mortality at 7 and 30 days after the first episode of candidemia was 13.4% and 25.2%, respectively. The overall in-hospital mortality rate was 35.1%. The treatment outcomes did not change over the study period. Multivariate analysis showed that delayed catheter removal (odds ratio [OR], 5.52; 95% confidence interval [CI]: 2.97–10.25), septic shock (OR, 5.49; 95% CI: 2.85–10.57), and breakthrough candidemia (OR, 3.66; 95% CI: 1.43–9.35) were independently associated with clinical treatment failure. In children with candidemia, underlying renal insufficiency and hematological/oncological malignancy, delayed catheter removal, and septic shock at onset were independently associated final in-hospital mortality. Analyzing the subgroup of non-neonatal children did not change the findings. We concluded overall mortality of pediatric candidemia remains high during the past decade. Prompt early catheter removal and aggressive treatment strategy in patients with septic shock would be critical to improve outcomes.

## Introduction

*Candida* bloodstream infection (BSI) is associated with high mortality and morbitidy rates among critically ill patients^1, 2^. The incidence of candidemia varies from 24 to 32.3 episodes/per 10,000 admissions^1, 3, 4^, and the mortality rate was 22–60%^2–6^. Recent strategies of antifungal prophylaxis or preemptive therapy for selected high-risk patients account for emergence of fluconazole resistant strains and an increased prevalence of non-*albicans Candida* species^7–9^. The mortality rate of children with candidemia remains high^10, 11^, and most patients have severe medical illness, surgical risk factors, presence of artificial devices, and frequently exposed to some high-risk medications^12–14^.

Numerous studies have described the epidemiology, clinical features, antifungal treatment and outcomes of children with candidemia^4, 10–16^. However, few researchers have investigated the reasons of treatment failure for children with candidemia^12–14^. The benefit of modifiable therapeutic strategies for candidemia is mainly derived from data and studies conducted in the adult settings^17–20^. Studies of pediatric candidemia were often limited by small sample sizes^4, 14^, lack of antifungal susceptibility testing^4, 13, 16^, or did not analyze the impact of different treatment strategies on outcome^4, 15, 16^. The aims of this study were to describe the clinical characteristics of *Candida* BSI in children, to present the results of antifungal susceptibility testing, and to assess the influences of therapeutic measures on the prognosis.

## Patients and Methods

### Study population, setting, and design

We included all hospitalized patients with age ≤18 years old in Chang Gung Memorial Hospital (CGMH) from January 2003 through December 2015, for whom ≥1 blood culture were positive for *Candida* species and who had symptoms and signs compatible with candidemia. Demographic characteristics, predisposing factors within the preceding 30 days from the onset of *Candida* BSI (defined as the day of first positive blood culture for *Candida* species), clinical management, and 30-day follow-up period were recorded in a dedicated database. The study was approved by the Institutional Review Board and Human Research Ethics Committee of CGMH, and a waiver of informed consent for anonymous data collection was also approved.

### Definitions

An incident episode of candidemia was defined as ≥1 positive blood culture drawn from a peripheral vein yielding a *Candida* isolate, with clinical symptoms and signs compatible with *Candida* BSI^2, 7, 16^. Episodes were considered to be separate if they occurred ≥1 month apart^21, 22^. Catheter-related *Candida* BSI was diagnosed when the catheter tip culture was positive for the same *Candida* species as those obtained from the peripheral vein and no evidence of infection at other site^18^. Severity of illness was measured by the Acute Physiology and Chronic Health Evaluation II (APACHE II) score on the day of candidemia and the presence of severe sepsis or septic shock at presentation^23^.

The primary study outcome was clinical treatment failure, which was defined according to the Mycoses Study Group and European Organization for Research and Treatment of Cancer consensus criteria^24^ as the following: (1) all-cause mortality between days 3 and 30 from the initial positive blood culture, or (2) persistent fungal BSI for ≥72 hours after the initiation of antifungal therapy. Patients who died within the first 72 hours were excluded from the analysis of outcome predictors to ensure that the potential impact of therapeutic strategies could be appropriately investigated. We also analyzed in-hospital all-cause mortality as secondary outcome.

### Microbiological Studies

All *Candida* isolates were processed to have species re-identification using Matrix-assisted laser desorption ionization time-of-flight mass spectrometry (MALDI-TOF, Bruker Biotype, software version 3.0, USA) and molecular methodology by sequencing the internal transcribed spacer regions (ITS1 and ITS2) from ribosomal DNA. Therefore, the identities of *Candida parapsilosis* sensu stricto, *Candida orthopsilosis* and *Candida metapsilosis* isolates were confirmed. *In vitro* antifungal susceptibilities of isolates were evaluated according to the EUCAST-Antifungal Susceptibility Testing microdilution method^25, 26^ Candida krusei* ATCC^®^ 6258 and *Candida parapsilosis* ATCC^®^ 22019 were used as the quality control strains for antifungal drug susceptibility testing.

### Statistical Analysis

Categorical variables were compared using the χ^2^ test or Fisher exact test, whereas student *t* test or Mann-Whitney *U* test were applied for continuous variables. All the significant tests were 2-tailed, with a P value less than 0.05 to be significant. We analyzed the impact of initial treatment strategy on the primary outcome with predictors for clinical failure assessed in the entire study population by using a backward stepwise logistic regression model.

For secondary outcome, the follow-up period was until death or discharge from hospital to evaluate variables related to death. A univariate logistic regression was fitted for each variable to test its relationship with mortality outcomes. Variables clinically relevant and statistically significant (P < 0.1) on univariate analysis were considered to build the multivariate regression model. Clinical interventions were maintained in the final model as a fixed variable. Potential confounders of treatment strategies (APACHE II score) were tested. Significant interactions between variables were ruled out. Statistical analyses were performed using SPSS, version 15.0 (IBM SPSS, Chicago, Illinois).

## Results

A total of 335 episodes of candidemia were identified during the study group. Of these, 16 case-patients were excluded because the *Candida* spp was unidentified (n = 5), and missing data regarding hospital courses (n = 4) and final outcomes (n = 7). Hence, this report is based on 319 episodes of candidemia identified in 262 pediatric patients. At the time of candidemia, 106 episodes (33.2%) occurred in the neonatal intensive care unit (NICU), 124 (38.9%) in pediatric ICU (PICU), 13 (4.1%) in burn-surgical ICUs, and 75 (23.5) in pediatric wards. The median length of hospitalization before Candida BSI was 29.0 days (IQR, 15.0–53.0 days). Baseline characteristics of the study population are outlined in Table 1.

**Table 1:** Demographic, baseline characteristics, clinical features and outcome of 319 episodes of *Candida* bloodstream infection in 262 pediatric patients.

Patients demographics	No. (%) (total n = 262)	Microbiological characteristics (total n = 319 episodes)	No. (%) (total n = 319)
Age		Pathogens	
Non-neonatal patients (years), mean ± SD	6.7 ± 4.5	*Candida albicans*	148 (46.4)
Neonatal patients (days), median (IQR)	22.5 (14.8–44.3)	*Candida parapsilosis*	86 (27.0)
Sex (male/female)	138 (52.7)/124 (47.3)	*Candida tropicalis*	18 (5.6)
Prematurity*		*Candida glabrata*	16 (5.0)
Birth body weight (g), median (IQR)	1006.4 (740–1610)	*Candida guilliermondii*	12 (3.8)
Gestational age (wks), median (IQR)	27.0 (25.0–31.5)	Other *Candida* spp.	39 (12.2)
Hospital days until diagnosis, median (IQR)	29.0 (15.0–53.0)	Source of BSI	
Patients source		Primary	201 (63.0)
Neonatal intensive care unit	94 (35.9)	Catheter-related BSI	70 (21.9)
Pediatric intensive care unit	95 (36.3)	Abdominal	31 (9.7)
Burn or surgical intensive care unit	10 (3.8)	Urologic	8 (2.5)
General wards	63 (24.0)	Lung	4 (1.3)
Underlying chronic comorbidities^#^		Meningitis	5 (1.6)
Congenital or genetic anomalies	28 (10.7)	Clinical presentation	
Neurological sequelae	89 (34.0)	Sepsis	307 (96.2)
Cardiovascular disease	22 (8.4)	Severe sepsis	127 (39.8)
Chronic lung disease and/or pulmonary hypertension	82 (31.3)	Septic shock	89 (27.9)
Gastrointestinal sequelae	71 (27.1)	Progressive and deteriorated^¶^	61 (19.1)
Renal sufficiency with/without dialysis	37 (14.1)	Disseminated candidiasis^$^	14 (4.4)
Hematological/Oncology cancer	42 (16.0)	Duration of candidemia, median (range)	3.0 (1.0–32.0)
Immunodeficiency	6 (2.3)	APACHE II score, mean ± SD	17.3 ± 4.2
Autoimmune disease	7 (2.7)	Predisposing risk factors^#^	
Hepatic failure or cholestasis	12 (4.6)	Receipt of systemic antibiotics^&^	298 (93.4)
Burn	5 (1.9)	Previous azole exposure^&^	34 (10.7)
Others^**^	2 (0.8)	Prior bacteremia^&^	160 (50.2)
Case years		Presence of CVC	310 (97.2)
2003–2006	82 (31.3)	Stay in an intensive care unit	244 (76.5)
2007–2011	98 (37.4)	Receipt of parenteral nutrition	217 (68.0)
2012–2015	82 (31.3)	Receipt of immunosuppressants	65 (20.4)
30-day all-cause mortality (from the first episode)	66 (25.2)	Artificial device other than CVC	160 (50.2)
Within 72 hours without receiving antifungal therapy	9 (3.4)	Prior surgery^&^	99 (31.0)
Early (>3 days, ≤7 days)	26 (9.9)	Neutropenia (ANC < 0.5 × 10^3^/μL)	80 (25.1)
Late (8–30 days)	31 (11.8)	Persistent BSI ≥ 72 hours of therapy	121 (37.9)
Overall final in-hospital mortality	92 (35.1)	*Candida* BSI attributable mortality	75 (23.5)
		Clinical treatment failure	141 (44.2)

*Only 94 patients from the neonatal intensive care unit.

^¶^Defined as candidemia episodes with more disseminated candidiasis and/or progressive multi-organ failure even after effective antifungal agents.

^#^Indicated the presence of underlying condition or risk factor at onset of *Candida* BSI, and most episodes occurred in patients with >1 underlying condition or risk factor.

^&^Within one month prior onset of invasive candidiasis.

**One patient had epidermolysis bullosa, and one patient had diabetes mellitus.

^$^Indicated positive *Candida* isolates recovered from more than two sterile sites, in addition to primary bloodstream infection.

APACHE II: Acute Physiology and Chronic Health Evaluation II score; BSI: bloodstream infection; ANC: absolute neutrophil count; CVC: central venous catheter; IQR: interquartile range; SD: standard deviation.

### Microbiological findings

There were three episodes where two different *Candida* species were obtained simultaneously on the same day and one episode where two different species were obtained on day 2 during the 30-day follow-up period. A bacterial pathogen was isolated in conjunction with *Candida* species in 31 episodes (9.7%) (the most common: gram-negative rods in 16, coagulase-negative staphylococci in 8, other gram-positive cocci in 13, and anaerobes in 2). Secondary candidemias were identified in a total of 48 (15.0%) episodes, with *Candida* isolates recovered from intra-abdominal space or abscess (n = 31), pleural fluid (n = 4), urinary source (n = 8), and cerebrospinal fluids (n = 5). Overall, 323 yeast strains were obtained from 262 episodes. Species distribution did not vary substantially between different ICU and general wards.

The results of *in vitro* susceptibility testing are summarized in Table 2. Overall, 86.1% of *Candida* isolates (254 of 295) were susceptible to fluconazole. Specifically, 97.0% *C*. *albicans* showed susceptibility, but 100% of *C*. *glabrata* (10 of 10) and 28.6% of *C*. *tropicalis* (4 of 14) were intermediate or resistant (minimum inhibitory concentration [MIC] ≥ 4 mg/L). Resistance to anidulafungin was uncommon: 1.2% for *C*. *parapsilosis* (1 of 86), 10.0% for *C*. *glabrata* (1 of 10), and no resistance among *C*. *albican* and *C*. *tropicalis*. However, the MIC_90_ of echinocandins against *C*. *parapsilosis* and *C*. *glabrata* was higher than those recorded for the most common Candida species. All isolates were susceptible to amphotericin B.

**Table 2:** Minimum Inhibitory Concentration Distributions Among Isolates of the *Candida* Bloodstream Infection in Children.

Pathogens/antifungals	No. of isolates with MIC (mg/L) of:											MIC (mg/L)		
	0.008	0.015	0.03	0.06	0.12	0.25	0.5	1.0	2.0	4.0	≥8.0	GM	MIC_50_	MIC_90_
*Candida albicans* (total n = 134 tested)														
Amphotericin B					1	10	117	6				0.484	0.5	0.5
Fluconazole					5	33	77	15			4	0.474	0.5	1.0
Voriconazole	104	24	2				1	1	2			0.010	0.008	0.015
Micafungin	80	31	19	3	1							0.119	0.008	0.03
Caspofungin	1	4	22	95	11	1						0.054	0.06	0.06
Anidulafungin		34	28	56	16							0.039	0.06	0.12
*Candida parapsilosis* (total n = 86 tested)														
Amphotericin B						6	51	28				0.598	0.5	1.0
Fluconazole					2	6	36	32	9			0.569	1.0	2.0
Voriconazole	19	39	21	6								0.017	0.015	0.03
Micafungin			1				15	49	18	2		1.015	1.0	2.0
Caspofungin			1		1	4	52	23	4			0.592	0.5	1.0
Anidulafungin			1	1		1	13	56	13	1		0.928	1.0	2.0
*Candida glabrata* (total n = 10 tested)														
Amphotericin B							4	6				0.757	1.0	1.0
Fluconazole											10	≥8.0	≥8.0	≥8.0
Voriconazole						1	6	2	1			0.615	0.5	2.0
Micafungin	2	7								1		0.023	0.015	4.0
Caspofungin			1	1	7						1	0.148	0.12	≥8.0
Anidulafungin			6	3					1			0.056	0.03	2.0
*Candida tropicalis* (total n = 14 tested)														
Amphotericin B							4	10				0.82	1	1
Fluconazole								3	7	3	1	5.123	4	8
Voriconazole					5	5	3				1	0.285	0.25	0.5
Micafungin		4	10									0.024	0.03	0.03
Caspofungin			2	7	2	3						0.081	0.06	0.25
Anidulafungin			2	4	6	2						0.089	0.12	0.25
Other *Candida* spp. (total n = 51 tested)														
Amphotericin B					3	21	17	7	3			0.412	0.5	1.0
Fluconazole					2	2	3	8	13	12	11	2.550	2	8
Voriconazole	7	4	6	17	10	4	1				2	0.060	0.06	0.25
Micafungin		7	4	5	2	11	16	5	1			0.180	0.25	1
Caspofungin			5	6	7	21	10				2	0.203	0.25	0.5
Anidulafungin		4	2	6	6	7	6	17	3			0.278	0.5	1

MIC: minimum inhibitory concentration.

MIC_50_ and MIC_90_: MIC required to inhibit 50% and 90% of the isolates, respectively.

GM: geometric mean.

### Clinical Data and Candidemia Management

Severe sepsis and septic shock were the clinical presentation of candidemia in 127 (39.8%) and 89 (27.9%) episodes, respectively. After antifungal therapy, 61 (19.1%) had progressively deteriorated course and 14 cases had positive *Candida* strains recovered from more than two non-blood sterile sites. Four had an obstructing renal fungus ball and one had septic thrombophlebitis during the follow-up period. Blood cultures were persistently positive for more than 3 days in 42.0% (134 of 319).

Overall, 310 episodes (97.2%) were treated with specific antifungal agents for candidemia (Table 3), including 45 (14.1%) episodes were breakthrough candidemia. All patients with candidemia were treated with standard dosing of antifungal agents. Antifungal therapy was initiated after a median of 2 days (range, 0–7) following the acquisition of the first diagnostic blood culture. The median duration of all antifungal therapy per episode was 17.0 days (range, 1–68). Of those 310 episodes for which an antifungal agent was used, 136 episodes (43.9%) had modification of the antifungal regimens during the treatment course. Nine cases never received targeted antifungal agents, and all of them died before the *Candida* isolates were documented in the blood cultures.

**Table 3:** Therapeutic approaches according to patient age.

Variable	Overall (total n = 319)	Non-neonatal episodes (>3 m) (total n = 213)	Neonatal episodes (≤3 m) (total n = 106)	P value^¶^
Initial antifungal therapy*				<0.001
Fluconazole/Voriconazole	210 (65.8)	154 (72.3)	56 (52.8)	
Amphotericin B	86 (27.0)	42 (19.7)	44 (41.5)	
Echinocandin-based regimen	14 (4.4)	11 (5.2)	3 (2.8)	
Final treatment regimens				<0.001
Fluconazole/Voriconazole	122 (38.2)	90 (42.3)	32 (30.2)	
Amphotericin B	94 (29.5)	47 (22.1)	47 (44.3)	
Echinocandin-based regimen	87 (27.3)	68 (31.9)	19 (17.9)	
Combination antifungal treatment	7 (2.2)	2 (0.9)	5 (4.7)	
No treatment	9 (2.8)	6 (2.8)	3 (2.8)	
Initiation of effective antifungal agents within 24 hours	131 (41.1)	97 (45.5)	34 (32.1)	0.021
Initiation of effective antifungal agents within 48 hours	221 (69.3)	156 (73.2)	65 (61.3)	0.033
Breakthrough candidemia	45 (14.1)	33 (15.5)	12 (11.3)	0.394
Central venous catheter (CVC) removal				
CVC removal within 48 hours	83/310 (26.8)	59/213 (27.7)	24/106 (22.6)	0.784
CVC removal within 72 hours	115/310 (37.1)	77/208 (37.0)	38/102 (37.2)	0.530
Elective CVC removal (after 72 hours of onset)	68/310 (21.3)	55/213 (25.8)	13/106 (12.3)	0.029
CVC removal after persistent candidemia	22/310 (7.1)	14/213 (6.6)	8/106 (7.5)	0.988

*Within the first 72 hours of administration of systemic antifungal antifungal drugs, we excluded a total of 9 episodes who never treated with antifungal agents before the patient died.

^¶^P values were the comparison between neonatal episodes and non-neonatal episodes.

As for CVC management, early CVC removal was performed within 48 hours and 72 hours after obtaining the first positive blood cultures in 26.8% (83 of 310) and 37.1% (115 of 310), respectively. Episodes with severe sepsis and septic shock were less likely to have early CVC removal (22.8% vs 44.8%, *p* < 0.001 and 22.5% vs 41.3%, *p* = 0.002, respectively). Elective CVC removal was done on 68 (21.3%) episodes, and 108 (34.8%) were treated with catheter *in situ*.

All cases of intra-abdominal candidiasis received surgical intervention. After effective antifungal therapy, 39.0% (121 of 310) had persistent fungemia for more than 72 hours. In total, 141 (45.4%) were considered as treatment failure based on our definition, and 22 of them had candidemia resolved only after CVC removal.

### Outcomes and Predictors of Mortality

Cumulative mortality at 7 and 30 days after the onset of candidemia was 13.4% (35 of 262) and 25.2% (66 of 262), respectively. Overall, the candidemia-attributable mortality rate was 23.5% (75 of 319) and in-hospital mortality rate for pediatric patients with candidemia was 35.1% (92 of 262). The treatment strategies and outcomes did not change greatly during the study period, when cases were divided into three study period (2003–2007, 2007–2011 and 2012–2015) and compared.

Candidemia caused by uncommon *Candida* spp. or non-albicans *Candida* spp. was associated with higher rate of treatment failure, so was delayed initiation of antifungal therapy (>48 hours) and underlying renal insufficiency (Table 4). However, after multivariate logistic regression, delayed CVC removal (odds ratio [OR], 5.52; 95% confidence interval [CI]: 2.97–10.25), septic shock (OR, 5.49; 95% CI: 2.85–10.57), and breakthrough candidemia (OR, 3.66; 95% CI: 1.43–9.35) were independently associated with clinical treatment failure.

**Table 4:** Univariate and multivariate logistic regression analysis of prognostic factors for clinical treatment failure*.

Variables	Univariate analysis			Multivariate analysis		
	Odds ratio	95% CI	P value	Odds ratio	95% CI	P value^#^
Patient category						
Neonates	1.20	0.75–1.91	0.451			
Children	1.0	(reference)				
Underlying chronic comorbidities						
Renal sufficiency with/without dialysis	3.11	1.53–6.33	0.002	1.99	0.86–4.60	0.109
Hematological/Oncological malignancy	1.42	0.75–2.67	0.284			
Septic shock	6.96	3.97–12.22	<0.001	5.49	2.85–10.57	<0.001
Delayed CVC removal (>72 hours)	6.40	3.69–11.07	<0.001	5.52	2.97–10.25	<0.001
Breakthrough candidemia	5.27	2.55–10.87	<0.001	3.66	1.43–9.35	0.007
Initial inadequate antifungal agents (first 24 hours)	1.32	0.81–2.18	0.275			
Delayed initiation of effective antifungal agents (48 hours)	1.78	1.13–2.82	0.014	1.14	0.61–2.13	0.673
Initial antifungal therapy						
Fluconazole/Voriconazole	1.0	(reference)				
Amphotericin B	0.682	0.41–1.15	0.147			
Echinocandin-based regimen	0.908	0.30–2.71	0.862			
Pathogens						
*Candida albicans*	1	(reference)		1	(reference)	
*Non-albicans Candida* spp.	1.72	1.09–2.71	0.021	1.42	0.78–2.57	0.254
Uncommon *Candida* spp.**	1.85	0.98–3.49	0.059	1.50	0.66–3.39	0.330
Case years						
2003–2006	0.665	0.38–1.15	0.148			
2007–2011	0.664	0.38–1.16	0.152			
2012–2015	1	(reference)				

APACHE II: Acute Physiology and Chronic Health Evaluation II score; CI: confidence interval; CVC: central venous catheter.

*All-cause mortality within day 3-30 (episodes with antifungal treatment) or persistent bloodstream infection for ≥72 hours from the initiation of antifungal therapy in 310 evaluable episodes of *Candida* BSI in children.

^#^Hosmer-Lemeshow *P* = 0.664.

**Uncommon *Candida* spp. included all *Candida* spp. in addition to *C*. *albicans*, *C*. *parapsilosis*, *C*. *glabrata*, *C*. *tropicalis*, *and C*. *krusi*.

We assessed predictors of candidemia-attributable mortality and final in-hospital mortality after episodes (cases) without antifungal treatment were excluded. Neonates had significantly higher of sepsis-attributable mortality and in-hospital mortality rates than children (32.1% vs 19.2%, *P* = 0.017 and 44.7% vs 29.8%, *P* = 0.022, respectively). On multivariate analysis (Table 5), underlying hematological/oncological malignancy, delayed catheter removal, breakthrough candidemia and septic shock at onset were independently associated with fungemia attributable mortality. Independent risk factors for in-hospital mortality in children with fungemia were underlying renal insufficiency with/without hemodialysis (OR, 4.69; 95% CI: 1.60–13.75) and hematological/oncological malignancy (OR, 4.41; 95% CI: 1.64–11.90), delayed catheter removal (OR, 2.13; 95% CI: 1.01–4.52), and septic shock at onset (OR, 15.64; 95% CI: 7.08–34.55). The significantly independent factor of delayed catheter removal and septic shock did not change after analyzing the patient subgroup of non-neonatal children (Supplemental Table 1).

**Table 5:** Multivariate logistic regression analysis for fungemia-attributable mortality and final in-hospital mortality in children with *Candida* bloodstream infection.

Variables	Fungemia attributable mortality (total episodes = 310, mortality n = 66)			Final in-hospital mortality* (total patients = 253, mortality n = 83)		
	Odds ratio	95% CI	P value^#^	Odds ratio	95% CI	P value^#^
Patient category						
Neonates	2.87	1.16–3.43	0.013	2.76	1.23–6.20	0.014
Children	1	(reference)		1	(reference)	
Underlying chronic comorbidities						
Renal sufficiency with/without dialysis	2.48	0.89–6.92	0.081	4.69	1.60–13.75	0.005
Hematological/Oncological malignancy	4.12	1.46–11.58	0.007	4.41	1.64–11.90	0.003
Septic shock	14.53	6.97–30.32	<0.001	15.64	7.08–34.55	<0.001
Delayed CVC removal (>72 hours)	2.95	1.24–7.03	0.015	2.13	1.01–4.52	0.049
Breakthrough candidemia	4.92	1.68–14.48	0.004	2.57	0.65–10.13	0.177
Delayed effective antifungal agents (>48 hours)	1.93	0.93–2.23	0.881	1.01	0.52–1.98	0.797
Final antifungal therapy						
Fluconazole/Voriconazole	1	(reference)		1	(reference)	
Amphotericin B	1.58	0.64–3.88	0.323	1.29	0.55–2.99	0.556
Echinocandin-based regimen	1.04	0.42–2.64	0.921	1.47	0.59–3.64	0.407
Combination regimens	1.26	0.78–4.74	0.375	11.26	1.21–105.2	0.034
Pathogens						
*Candida albicans*	1	(reference)		1	(reference)	
*Non-albicans Candida* spp.	1.14	0.68–1.93	0.622	1.07	0.49–2.32	0.868
Uncommon *Candida* spp.	1.40	0.69–2.85	0.354	1.40	0.59–2.78	0.983
Case periods						
2003–2008	0.99	0.52–1.87	0.97			
2007–2011	1.78	0.56–2.09	0.82			
2012–2015s	1	(reference)				

CI: confidence interval; CVC: central venous catheter.

*For patients with more than two episodes of *Candida* bloodstream infection, data from the first episode of *Candida* bloodstream infection were enrolled into the model of multivariate analysis for predictors of final in-hospital mortality.

^#^Hosmer-Lemeshow *P* = 0.649 and 0.427 for fungemia attributable mortality and in-hospital mortality, respectively.

## Discussion

There is relatively scarce information regarding the impact of specific therapeutic strategies on an adverse outcome of pediatric candidemia^6, 27, 28^, as the authors of recent cohort studies did not analyze the influence of illness severity, catheter removal and timing of antifungal treatments^11, 28^. Furthermore, these studies did not consider the effectiveness of antifungal agents based on *in vitro* antifungal susceptibility, and the second or recurrent episodes were often ignored^4, 10, 11, 28^. After multivariate analysis, we found early catheter removal, breakthrough candidemia, and septic shock at onset independently associated with treatment failure of candidemia in children.

The 30-day mortality of our cohort was slightly higher than previous pediatric studies^4, 14, 28^, which reported a mortality rate between 11.4% and 44%^4, 10, 11, 14, 27, 28^. The higher mortality rate in our patients with candidemia could be explained by the higher rates of severe sepsis and septic shock in our cohort. While septic shock has been found the independent risk factor of final mortality in the adult studies^6, 18, 29^, this issue was rarely mentioned in the pediatric population^4, 10, 11, 14, 27, 28^. Accordingly, septic shock at infection onset seems a clear surrogate marker for illness severity and emerged as independently associated with poor outcome in our cohort, since no universal illness scoring system can be applied simultaneously on both neonates and children.

The impact of CVC management on the outcome of candidemic patients have been extensively investigated^22, 30–33^. While recent multicenter studies did not find significant impact of early CVC removal on outcome in either neonates or children^4, 11, 28^, we documented delayed CVC removal not only exerted a significant effect on the odds of clinical failure but also was associated with prolonged candidemia and final in-hospital mortality. However, whether prompt catheter removal contributes significantly to outcome remains a debatable issue because there are no randomized controlled trials, either in the adult or pediatric settings, to document the benefit when the source of candidemia is not the catheter. The conflicting conclusions of previous studies most likely resulted from different study designs and failure of controlling the bias of illness severity^11, 22, 29–33^, especially as there might also be selection bias in their hospital. Nonetheless, based on our findings and the expert guidelines^34, 35^, we believe that CVC removal should be attempted in children with candidemia when feasible.

Several studies concluded echinocandin and anidulafungin treatments were associated with decreased treatment failure^30, 36, 37^, other studies had conflicting conclusions and found adequate source control and timely antifungal treatment were independently associated with greater risk of mortality^6, 11, 18, 29^. In our cohort, we found antifungal regimens did not have significant influence on treatment outcomes, which were significantly associated with early catheter removal, underlying comorbidities and severity of illness. In our cohort, very few patients received echinocandins as their initial treatment, which may be due to the fact that most *Candida* isolates of candidemia in children were susceptible to fluconazole or amphotericin B. Another possible cause of prolonged candidemia in patients who had retained catheter may be biofilm formation^38, 39^. In this situation, the recently published European guidelines recommend use of echinocandin or liposomal amphotericin B as the therapeutic regimen, which have *in vitro* activity against biofilms^40^.

The presence of underlying chronic comorbidities and distributions of *Candida* species were supposed to affect the treatment outcomes^30, 41, 42^. In consistent with other studies in the pediatric population, our cohort did not include enough patients to support a worse outcome due to different *Candida* species^10, 11, 14, 28^. We found renal failure and hematological/oncological malignancy independently associated with final in-hospital mortality but not clinical treatment failure, which suggested these patients died of major organ failure after clearance of candidemia and could account for the high mortality rate of our cohort.

The proportion of fluconazole-resistant strains (14.0%) in our cohort was slightly higher than previous pediatric studies^11, 43^. However, antifungal resistance cannot explain the significantly higher rate of persistent fungemia after effective antifungal therapy^9, 18, 41^. Additionally, our cohort had higher rates of retained CVC when compared with other studies^18, 41^, which may be due to more patients had severe sepsis and septic shock and early CVC removal was not possible. Based on our results, prompt early catheter removal and aggressive treatment strategies in patients with septic shock would be critical to improve patient outcomes.

The issue of breakthrough candidemia was rarely mentioned in the pediatric studies, and previous reports concluded breakthrough candidemia mainly occurred in acutely ill patients, those with serious comorbid conditions, frequently neutropenic or previously treated with corticosteroids or other immunosuppressive drugs^22, 44–46^. In our cohort, breakthrough candidemia were mainly caused by non-*albicans* Candida species (73.3%), and in patients with neutropenia (44.4%), underlying hematological/oncological malignancy (24.4%) and under immunosuppressants (28.9%). In our cohort, most isolates of breakthrough candidemia were fluconazole susceptible, but it was found independently associated with treatment failure and mortality. Therefore, the higher treatment failure rate of breakthrough candidemia might be due to the severe underlying illness, and echinocandins are suggested^22^.

There were some limitations in this study. This is a single center, retrospective study. Therefore, our observations may be less applicable to different institutions when compared with multicenter, prospective studies. We did not perform serial blood cultures follow-up systemically in every episodes, and the treatment strategies were not controlled by bias of illness severity. Furthermore, sample size limitations precluded any subgroup analysis according to the specific non-*albicans Candida* species as the pathogen.

In conclusion, we identified that delayed catheter removal, breakthrough candidemia and septic shock were the most important predictors of treatment failure. Underlying renal failure and hematological/oncological malignancy contribute significantly to candidemia-attributable mortality. The treatment outcomes did not improve over the past decade. Therefore, prompt early catheter removal and aggressive treatment strategies in patients with risk factors for adverse outcomes would be important.

## Electronic supplementary material

Supplementary Table 1
