# Supplementary material for: Clinical and microbiological characteristics, and impact of therapeutic strategies on the outcomes of children with candidemia
Source: Sci Rep. 2017 Apr 24;7:1083. doi: 10.1038/s41598-017-01123-6 (PMC5430948; doi:10.1038/s41598-017-01123-6)
Supplement: Supplementary file 1 — Supplementary Table 1 [file 41598_2017_1123_MOESM1_ESM.doc]

Clinical and microbiological characteristics, and impact of therapeutic strategies on the outcomes of children with candidemia

Ming-Horng Tsai, MD^1,4,6^; Jen-Fu Hsu, MD^2,4^; Shih-Ming Chu, MD^2,4^; Pey-Jium Chang PhD^6^; Mei-Yin Lai, MD^2,4^; I-Hsyuan [Wu](https://tw-mg31.mail.yahoo.com/neo/b/compose?to=shaohung.wang@gmail.com) MD^2,4^; Hsuan-Rong Huang, MD^2,4^; Ming-Chou Chiang, MD^2,4^; Ren-Huei Fu, MD PhD^2,4^; and Jang-Jih Lu, MD, PhD^3,5*^

Supplemental table 1. Multivariate logistic regression analysis for clinical treatment failure in non-neonatal children with *Candida* bloodstream infection

| Variables | Clinical treatment failure | | |  | Fungemia-attributable mortality | | |
| --- | --- | --- | --- | --- | --- | --- | --- |
|  | Odds ratio | 95% CI | P value* |  | Odds ratio | 95% CI | P value* |
| Underlying chronic comorbidities |  |  |  |  |  |  |  |
| Renal sufficiency with/without dialysis | 1.71 | 0.68-4.26 | 0.254 |  | 2.94 | 0.86-9.99 | 0.085 |
| Hematological/Oncological malignancy | 1.29 | 0.61-2.76 | 0.510 |  | 3.74 | 1.38-10.15 | 0.010 |
| Septic shock | 2.93 | 1.35-6.37 | 0.007 |  | 10.26 | 4.09-25.76 | < 0.001 |
| Delayed CVC removal (> 72 hours) | 4.23 | 2.10-8.51 | < 0.001 |  | 2.14 | 1.04-4.82 | 0.043 |
| Breakthrough candidemia | 2.84 | 1.02-7.86 | 0.045 |  | 2.41 | 0.67-8.67 | 0.177 |
| Delayed effective antifungal agents (> 48 hours) | 1.38 | 0.66-2.86 | 0.391 |  | 0.58 | 0.17-1.95 | 0.376 |

CI: confidence interval; CVC: central venous catheter

*Hosmer-Lemeshow *P* = 0.740 and 0.141 for clinical treatment failure and fungemia attributable mortality, respectively.
